# Sudden respiratory and circulatory collapse after cesarean section: Amniotic fluid embolism or other reasons? – a case report

**DOI:** 10.1186/s12884-022-04701-3

**Published:** 2022-04-28

**Authors:** Jinxi Zhang, Chao Yu, Hui Liu, Qing Zhu

**Affiliations:** Department of Anesthesiology, West China Second University Hospital; Key Laboratory of Birth Defects and Related Diseases of Women and Children (Ministry of Education), Sichuan University, Chengdu, China

**Keywords:** Cardiopulmonary collapse, Amniotic fluid embolism, Anaphylaxis, Antibiotics, Case report

## Abstract

**Background:**

For a healthy parturient, a cardiopulmonary collapse that suddenly occurs shortly after an uneventful caesarean section is a relatively rare event and presents a significant challenge for the anesthesia provider.

**Case presentation:**

Amniotic fluid embolism (AFE) is characterized by acute and rapid collapse and is well known to the obstetric team. Our patient experienced sudden cardiovascular collapse, severe respiratory difficulty and hypoxia, in the absence of other explanations for these findings at the time, and thus AFE was immediately become the focus of the consideration. However, there is no quick, standard laboratory test for AFE, therefore the diagnosis is one of exclusion based on presenting symptoms and clinical course. After given symptomatic treatment, the patient made an uneventful initial recovery in a short period and developed a rash. We recognized that the postpartum shock was associated with delayed anaphylaxis of antibiotics.

**Conclusions:**

These observations have implications for understanding whenever administering drugs in surgery, which may affect the anesthesiologist’s judgment regarding the complications of anesthesia. Even though serious complications of common perioperative drugs may rarely occur, anesthesia providers should be aware of the consideration. Early recognition and effective treatment are more important than prompt diagnosis.

## Background

Sudden respiratory and circulatory collapse after cesarean section is a clinical condition that may lead to poor maternal outcome. Because a number of clinical events in pregnancy may be responsible for cardiopulmonary collapse, sometimes, it is difficult to make a timely exact diagnosis. Importantly, establishment of appropriate supportive treatment is required. We present a healthy parturient suffering from unexplained cardiopulmonary collapse after an uneventful cesarean section and briefly discuss the more common reasons and the management for the clinical settings.

## Case presentation

A 25-year-old nulliparous woman with a single pregnancy was admitted to the labor and childbirth service of our hospital at 40 weeks 4 days’ gestation for term prelabor rupture of the membranes. The patient’s previous medical history was unremarkable. Emergency cesarean section was scheduled for the parturient because of retention of fetal head descending and persistent occipito-posterior position.

**Fig. 1:**
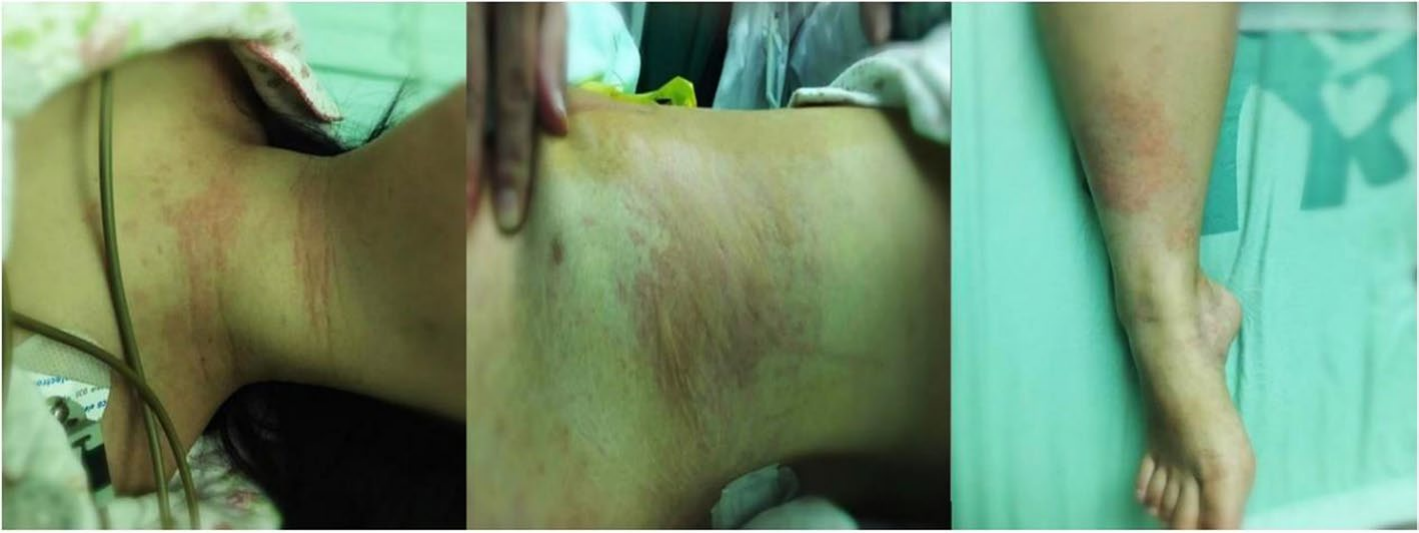
Skin rashes on the patient’s neck, waist and limbs

On arrival at the operating room, the parturient without premedication was in slightly anxiety, but the vital signs and oxygen saturation were normal. The nursing team placed catheter for fluid administration— a large-bore peripheral intravenous infusion catheter— followed by infusion of 500 ml of lactated Ringer's solution prior to anesthesia. Combined spinal-epidural anesthesia was administered in lateral decubitus at the L3-L4 interspace, with 2.5 ml of 0.5% isobaric bupivacaine. The sensory blockade reached the level of T6. Cesarean section was uneventful. The duration of surgery was 38 min, with an estimated blood loss of 300 ml. Nevertheless, two minutes after the end of cesarean section, the patient coughed suddenly, her systolic blood pressure fell to 60 to 70 mm Hg, oxygen saturation to 60 to 70%, and the pulse increased to 110 to 120 beats per minute. The patient complained of dyspnoea, and her lips and nails became cyanotic; auscultation of the chest revealed bilateral scattered wheezing. The mask ventilation with 100% oxygen was applied, and 200 mg hydrocortisone was injected intravenously. In the meanwhile, metaraminol, epinephrine and intravenous fluids were administration. Approximately 20 min later, vital signs returned to normal range, cyanosis disappeared and lung sounds were slightly coarse. The patient was transferred to the intensive care unit. At this moment, we observed skin rashes on her neck, waist and limbs (Fig. 1). The post-partum echocardiogram and coagulation testing revealed no abnormalities.

## Discussion and Conclusion

After an uncomplicated caesarean section at term, this healthy parturient had a sudden cardiorespiratory collapse. An unexpectedly high level of neuraxial anesthesia need to be considered as a cause of intraoperative cardiorespiratory collapse. However, in this case, so much time had passed since the injection of local anesthetics, and subsequent events seemed unlikely to be directly related to an anesthesia complication. Moreover, the parturient was very young and had no history of heart disease, and the intraoperative electrocardiography did not suggest arrhythmias, myocardial ischemia and infarction. Therefore, cardiovascular events were also excluded.

Amniotic-fluid embolism (AFE), although extremely rare, is well known and dreadful for obstetricians, and because it seemed the most likely explanation for this patient’s condition at the time of the cardiopulmonary collapse, this diagnosis will immediately become the focus of the consideration. AFE is characterized by acute and rapid collapse, that affects pregnant women shortly before, during, or immediately following labor and childbirth, as a result of amniotic fluid or fetal material entering the maternal circulatory system [1]. The rate at which it occurs is approximately 1 in 40,000 births and the estimated mortality (death) rate is 11–44% [2, 3]. The typical presentation of AFE includes two phases. A first phase is marked by acute respiratory distress and cardiovascular collapse. This is followed by a second phase which is characterised by massive hemorrhage, disseminated intravascular coagulopathy (DIC) or consumptive coagulopathy develops [4]. Unfortunately, there is no quick, standard laboratory test for AFE, therefore the diagnosis is one of exclusion based on presenting symptoms and clinical course, very often on postmortem report [5]. As in this patient, sudden onset of dyspnea in the face of cardiovascular collapse should lead the clinician to suspect AFE and initiate aggressive and supportive treatment. However, there was no evidence of bleeding. After a short period, she made an uneventful initial recovery. Particularly, later on, the patient developed a rash, which indicated the presence of other cause for the sudden onset of cardiorespiratory compromise. Then, AFE should be overdiagnosed.

We recognized that the clinical course and hemodynamic changes were more similar to patients with anaphylactic shock. Anaphylaxis occurring during pregnancy is a clinical condition that can be life threatening and almost always an unanticipated reaction [6]. Little is known about the epidemiology of immediate allergic hypersensitivity occurring during childbirth, however, the exact incidence is probably underestimated. For a susceptible pregnant woman, any agent can potentially trigger anaphylaxis [7]. Nevertheless, antibiotics, prophylactic administration for decreasing the incidence of postpartum uterine infection and wound infection, are the most commonly reported triggers of anaphylaxis during caesarean section [8]. In the case, the patient with no history of anaphylaxis and in an emergency cesarean section of no time to skin test, as our institutional general rule, intravenous clindamycin was used after clamping of the umbilical cord, which appeared to be the main culprit agents.

The hallmarks of anaphylaxis are cardiovascular collapse, bronchospasm, and cutaneous–mucous signs [9]. The classic symptoms usually occur within minutes of exposure to an allergen. Sometimes, however, it can occur a half-hour or longer after exposure. Thus, sudden respiratory and circulatory collapse usually represent the inaugural event. The subcutaneous vascular bed is susceptible to vasoconstrictive influences on the early stage of anaphylaxis, and the absence of mucocutaneous sign or symptom does not exclude the diagnosis. Ultimately, mucocutaneous signs may appear after the normalization of the arterial blood pressure [9, 10]. It is more important to recognize the signs and symptoms rapidly and treat effectively than to diagnosis, a basic principle similar to that in AFE. The management rules for the treatment of an anaphylactic attack during pregnancy include (1) withdraw the suspected culprit agent and immediately call for help, (2) maintain airway with 100% oxygen and administer early intravenous epinephrine, (3) before childbirth, position the patient in left lateral uterine displacement and make a plan for emergency caesarean section [6].

Currently, the ACOG has recommended the prophylactic administration of an antibiotic within 1 h of the start of caesarean section [11]. Because of concern that adverse effects of fetal antibiotic exposure, the optimal timing of antibiotic administration (before skin incision versus after clamping of the umbilical cord) remains controversial. The use of antibiotics may affect the anesthesiologist’s judgment regarding the administration and complications of anesthesia. Whenever administering perioperative antibiotics, even though serious complications may rarely occur, anesthesia providers should be aware of the consideration.

Sudden respiratory and circulatory collapse may be catastrophic to both mother and fetus during caesarean section. A carefully obtained medical history, meticulous observation of the patient, and monitoring of vital signs usually provide early warning of an impending reaction. Early recognition and effective treatment are more important than prompt diagnosis, and are necessary to reduce associated morbidity and to avoid mortality.

## Data Availability

The datasets used and/or analysed during the current study available from the corresponding author on reasonable request.
